# The effects of *Carthamus tinctorius* L. on placental histomorphology and survival of the neonates in mice

**Published:** 2012

**Authors:** Ali Louei Monfared, Amir Parviz Salati

**Affiliations:** 1 *Department of Basic Sciences, Faculty of Para-Veterinary Medicine, University of Ilam, Ilam, I. R. Iran*; 2 *Department of Fisheries, Faculty of Marine Natural Resources, Khorramshahr University of Marine Science and Technology, Khorramshahr, I. R. Iran*

**Keywords:** *Carthamus tinctorius*, Histomorphology, Mice, Placenta, Prenatal toxicity

## Abstract

**Objective:** *Carthamus tinctorius* L. (Safflower) is a member of the asteraceae family which is used as a food additive but also has medicinal applications. This study investigated its effects on placental histomorphology and survival of mice neonates.

**Materials and Methods: **Eighty Balb/C pregnant mice were randomly distributed into one control and three experimental groups (n=20). The control group received only distilled water, whereas experimental groups were administered intraperitoneally *C. tinctorius* extract at doses of 0.7, 1.4, and 2.8 mg/kg during the organogenesis period (6^th^ to 16^th^ day of gestation). On the 17^th^ day of gestation, half of the animals were euthanized; their fetuses and placentas were removed and histomorphological study was performed. In the rest of the animals after parturition, the number of neonates was counted. Survival rates were periodically calculated for neonates within days 5, 15, 25, and 42 after birth. The results were evaluated by one-way ANOVA.

**Results:** The results showed that treatment with 1.4 and 2.8 mg/kg* C. tinctorius* extract caused reduction in the trophoblastic giant cells ratio and increasing in the proportion of labyrinthine interhemal membrane (LIM). Moreover, the size of the labyrinthine zone per whole placenta, weight, diameter, and thickness of the placenta in the mice administered with 1.4 and 2.8 mg/kg* C. tinctorius* extract became lower than those of controls (p<0.05). In addition, in the treated mice with 1.4 and 2.8 mg/kg* C. tinctorius* extract, the number of neonate was drastically decreased on days 5, 15, 25, and 42 after birth.

**Conclusion:** It is concluded that treatment with *C. tinctorius *extract in doses of 1.4 and 2.8 mg/kg induces toxic changes in the placental structure so caution should be paid to popular consumption of this plant both as an alternative medicine and as a food additive.

## Introduction

The plant *Carthamus tinctorius* (Safflower) is a member of the asteraceae family that is cultivated mainly for its seed, which is processed to edible oil and used as bird seed. In addition, its flowers have applications in medicine and food industry (Elias et al., 2002 ▶; Mass, 1986; ▶ Siddiqi et al., 2009 ▶). Its extract has been reported to be useful in the treatment of cardiomyopathy (Tien et al., 2010), gynecological diseases (Zhang et al., 1998 ▶), menstrual problems (Wang and Li, 1985), as anti-inflammatory (Jun et al., 2011), and antitumor agents (Loo et al., 2004 ▶) in traditional medicine. 

It has been shown that paternal exposure to some herbal plants could result in adverse outcomes on the survival and health of the neonates (Macías-Peacok, 2009 ▶; Nordeng and Havnen, 2004 ▶). Moreover, Nobakht et al. (2000) reported an association between maternal exposure to *C. tinctorius* extract at doses of 1.2 and 2 mg/kg and congenital malformations in their offspring. Virtually, all survey data agree that users of herbal medicine products including *C. tinctorius *are predominantly female (Eisenberg et al., 1998 ▶). Therefore, one might assume that pregnant women frequent use of herbal medicine products are often perceived as being “natural and therefore free of risks” (Ernst, 2002 ▶; Eisenberg et al., 1998 ▶).

The placenta, in addition to its myriad of functions during development, is recognized as a target for the toxic actions of some materials (Foster et al., 2008 ▶). Furthermore, foreign compounds may interfere with placental function at many levels and any deviation from normal development may constitute a potential threat to placental function, resulting in preterm delivery, congenital malformation, or abortion (Myllynen et al., 2005 ▶). Since there is not enough data about effects of *C. tinctorius* on placental structure and neonate survival changes, this study was done.

## Materials and Methods

*Carthamus tinctorius* (Safflower or Golrang) plants were purchased from Emam-Reza medicinal plants market (Ilam, Iran) and botanical identification was confirmed at the herbarium of Ilam University (Exsiccatae number: 637-2-90). For extraction preparation, the plant material was washed with sterile water, dried in shade at room temperature for 2 weeks and ground in an electric mill to obtain particles smaller than 4 mm. This material was extracted by maceration in 70% methanol solution at 50 ^◦^C for 2 hours. The extract was filtered through a Wattman ≠1 paper and evaporated to dryness in a rotary evaporator under reduced pressure. The dried material was stored under refrigeration at 4-8 ^◦^C until its use. 

In order to do the experiments, 80 male and 80 female Balb/C mice at 30 grams of initial body weight and aged 10 weeks were purchased from Razi Institute (Karaj, Iran). The animals were housed in a controlled environment (temperature of 23±1 ºC; relative humidity 45±5%; 12:12 h light-dark natural cycle) and had ad-lib access to drinking water and food. Mice were allowed to be acclimatized to the laboratory environment at least 6 days before commencement of testing. For mating, one female was placed into cage of one male overnight (12 h). Day 0 gestation was determined by a sperm-positive vaginal smear following copulation. The pregnant animals were randomly distributed into one control group and three experimental groups, each comprising of 20 mice. The control group received only distilled water, whereas experimental groups were administered intraperitoneally *C. tinctorius* extract at doses of 0.7, 1.4 and 2.8 mg/kg/day during the organogenesis period (6^th^ to 16^th^ day of gestation). The doses were determined on the basis of a primary study. 

On the 17^th^ day of pregnancy, half of the animals (n=40) were anesthetized, the abdomen cavity was opened and placental/fetal units were carefully removed from the uterus. For optical microscopy, the placenta immersion was maintained overnight in Bouin^´^s solution for fixation. Afterward, the placentas were sectioned at 5-µm and stained with Haematoxylin and Eosine (H&E). The sections were photographed directly using a digital camera (COOLPIX 950, Nikon, China) which was connected to a light microscope and computer. 

In all groups, the number of different placental cells was counted by manual counting of at least 100 cells in four randomly chosen microscopic fields per placenta. In addition, the size of different parts of the placental tissue was analyzed using Image Tool® 3.0 software (UTHSCSA, San Antonio, TX, USA) and compared between experimental and control groups. For morphological study, the weight of the placentas was measured manually using an electronic digital scale (Tanita Corporation, Tokyo, Japan). In addition, the diameter and thickness of the placentas were determined manually using a caliper (Mitutoyo, Kanagawa, Japan). 

In the rest of the animals (n=40), after parturition, the number of neonates was counted in each group. Survival rates were periodically calculated for neonates within days 5, 15, 25, and 42 after birth using the following formula: the number of living neonates/the number of dead neonates (Tachibana et al., 2007 ▶). All procedures were carried out in accordance with institutional guidelines for animal care and use at the University of Ilam. The study was approved by the institutional animal ethical committee (Ref. No: IAEC/12/09/IU/2010).

All results were expressed as mean±SEM. The analysis of variance (ANOVA) was used to test the overall significance of differences among the means. Tukey- Kramer´s Multiple Comparison Test was applied for *post-hoc* comparison. Computations were performed using site-licensed SPSS statistical software (SPSS, Chicago, IL, USA). A probability level of less than 5% (p<0.05) was considered as significant. 

## Results

**Figure 1:**
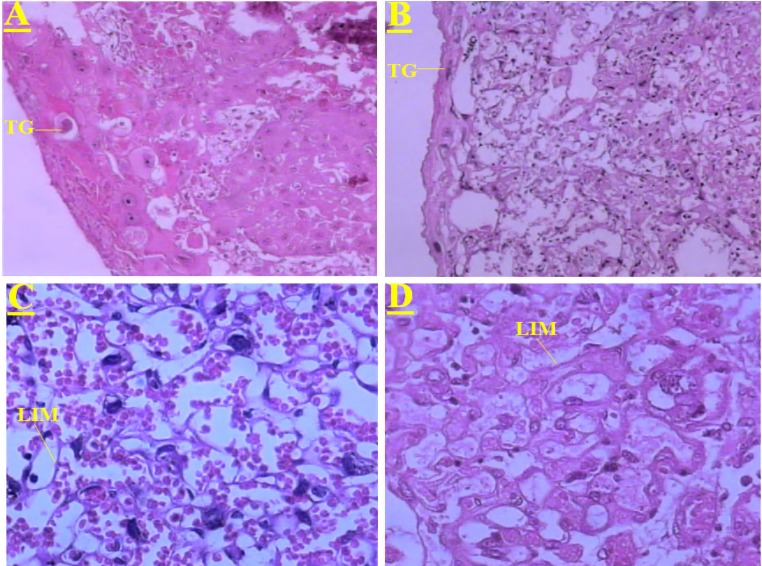
(A): Transverse section through the placenta of control mice. This part shows normal proportion of the trophoblastic giant cells (TG) in the placenta. (B): Transverse sections through the placenta of the mice treated with *C. tinctorius* extract at dose of 1.4 mg/kg/day. This part shows a decrease in the trophoblastic giant cells (TG) ratio in the placenta. (C): Transverse section through the placenta of the control animals. This part shows normal thin diameter of labyrinth interhemal membrane (LIM) in the placenta. (D): Transverse section through the placenta of the mice treated with *C. tinctorius* extract at dose of 1.4 mg/kg/day. This part shows an increase in the diameter of the labyrinth interhemal membrane (LIM) in the placenta. (Haematoxylin and Eosine stain) (A, B: × 100 and C, D: × 400).

**Figure 2:**
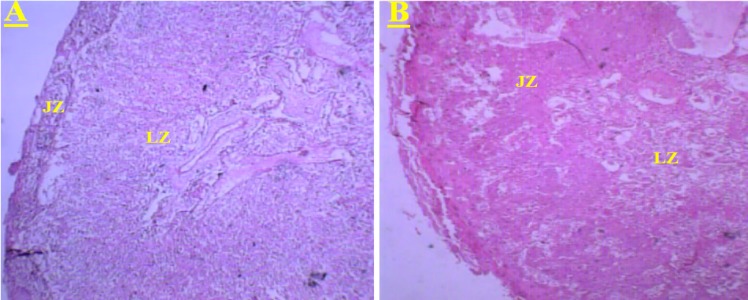
(A): Transverse section through the placenta of control animals. This part shows the normal proportions of the labyrinth (LZ) and junctional zones (JZ) of placenta. (B): Transverse section through the placenta of the mice treated with *C. tinctorius* extract at dose of 2.8 mg/kg/day. This part shows decreasing in the size of the labyrinthine zone (LZ) and increasing in the size of the junctional zones (JZ) per whole placenta. (Haematoxylin and Eosine stain) (A, B: × 100).

**Figure 3:**
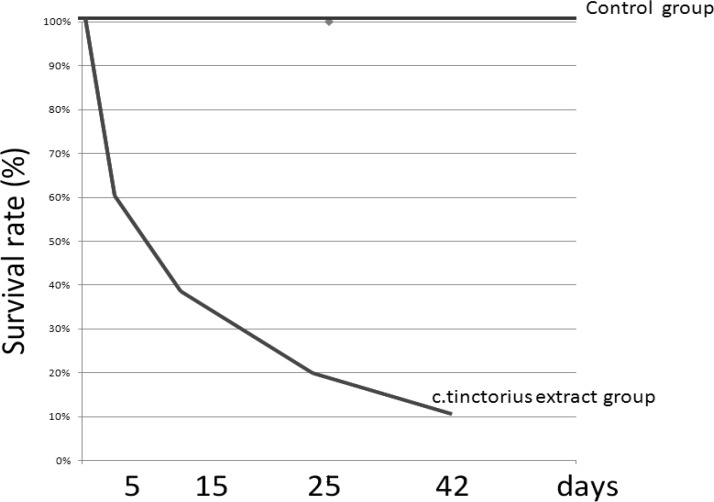
Survival rates profile of the neonate after birth. In the mice treated with *C. tinctorius* extract, a drastic decrease was recognized in these rates on days 5, 15, 25, and 42 after birth

The effects of *C. tinctorius* extract on the histological changes of the placenta are shown in Figures 1 and 2. These changes included decrease in the number and size of trophoblastic giant cells and increase in the diameter of labyrinthine interhemal membrane (LIM) in the treated mice with 1.4 and 2.8 mg/kg extract in comparison with the control animals. Moreover, administration of *C. tinctorius* extract at doses of 1.4 and 2.8 mg/kg could decrease the proportion of the labyrinthine zone per whole placenta (Figure 2). Additionally, in all the extract-treated animals, morphological changes including significant lower weight and diameter of the placenta were seen (p<0.05) (Table 1). Although the thickness of the placenta in the extract treated mice was decreased in a dose-response pattern, but there were not a significant statistical difference between three experimental groups (Table 1). In the treated mice with 1.4 and 2.8 mg/kg *C. tinctorius* extract, and the number of neonates was drastically decreased on days 5, 15, 25 and, 42 after birth. The survival rate of the control neonates was 100% within 42 days after the birth (Figure 3).

**Table 1:** Mean±SEM. of the morphological parameters of the placenta in control and treated mice with different doses of *C. tinctorius* extract

Parameters/Groups	Control	0.7 mg/kg/day * C. tinctorius*	1.4 mg/kg/day * C. tinctorius*	2.8 mg/kg/day * C. tinctorius*
Placental weight (g)	0.45 ± 0.03 *^ a^	0.22± 0.03^ b^	0.26± 0.07^ b^	0.19± 0.09^ b^
Placental diameter (mm)	7.16± 0.01^ a^	6.1± 0.07^ b^	6.28± 0.06^ b^	6.51± 0.09^ b^
Placental thickness (mm)	1.07± 0.02^ a^	0.08± 0.06^ b^	0.05± 0.01^ b^	0.04± 0.06^ b^

* Significant differences with the control group as p<0.05

## Discussion

Our findings showed that administration of 1.4 and 2.8 mg/kg *C. tinctorius* in mice could produce significant changes in the placental structure and the neonate viability. In the present study, the placental trophoblastic giant cells in the treated mice with 1.4 and 2.8 mg/kg *C. tinctorius* extract were poorly developed. It is suggested that trophoblastic giant cells participate in a number of processes essential to a successful pregnancy including blastocyst implantation, remodeling of the maternal deciduas, and secretion of hormones that regulate the development of both the fetal and maternal compartments of the placenta (Ogren, 1988 ▶). Furthermore, trophoblastic giant cells control the maternal blood flow (Dong et al., 2003 ▶) and their angiogenic potential is important for successful placentation (Gagioti et al., 2000 ▶; Abbot and Buckalew, 2000 ▶). 

Similar results have been reported by Nobakht et al. (2000) and Kosif et al. (2010). The diameter of the LIM is a critical parameter to placental physiology (Coan, 2004 ▶), thus, in the present study, significant thickening of LIM in the placentas of *C. tinctorius* treated mice, confirming an increased diffusion distance between the maternal and fetal blood. These changes are associated with reduced nutrient supply from the maternal to the fetal circulation (Laurie et al., 1992 ▶; Masashi et al., 1992 ▶). As showed in this study,* C. tinctorius *at doses of 1.4 and 2.8 mg/kg could significantly reduce the proportion of the labyrinthine zone per whole placenta, weight, diameter, and thickness of the placenta. These findings revealed that *C. tinctorius *may cause poor formation of the placenta which probably leads to intrauterine growth retardation. Similar findings have been reported by Nana et al. (2008), Kosif et al. (2010), and Monteiro et al. (2001).

In the present study, analysis of the placentas belonging to the mice treated with 1.4 and 2.8 mg/kg extract revealed that formation of the labyrinthine layer of the tissues that eventually establishes the embryonic and maternal vascular interchange was disturbed (Figures 1 and 2) when compared with placentas from control animals. The abnormal vessel formation in labyrinth zone might be caused by reduced functional capacity of the trophoblastic barrier after C*. tinctorius* exposition. These results are in accordance with those obtained by Kosif et al. (2010) and by Nobakht et al. (2000). 

Considering the above-mentioned observations, it is concluded that treatment with 1.4 and 2.8 mg/kg *C. tinctorius *extract induces abnormal changes in the placental structure. These changes probably disrupt placental functions and leads to an elevation in the mortality of the neonates. Although mechanism(s) of *C. tinctorius* toxic effects on the placentas and neonates is not clear but the leaf of this plant contains different active components including flavonoids, glucosides, and rutinosides (Li Fan et al., 2009 ▶) that could be responsible for these changes. Further studies will be needed to explore exact causative factors for *C. tinctorius*-induced placental and neonate toxicity. The present findings of *C. tinctorius*-induced life-threatening placental changes and neonate survival in mice suggest that caution should be paid to popular consumption of this plant both as an alternative medicine and as a food additive.
